# Draft Genome Sequence of an Invasive *Streptococcus agalactiae* Isolate Lacking Pigmentation

**DOI:** 10.1128/genomeA.00015-16

**Published:** 2016-02-25

**Authors:** Pallavi Singh, David M. Aronoff, H. Dele Davies, Shannon D. Manning

**Affiliations:** a Department of Microbiology and Molecular Genetics, Michigan State University, East Lansing, Michigan, USA; b Department of Medicine, Vanderbilt University, Nashville, Tennessee, USA; c University of Nebraska Medical Center, Omaha, Nebraska, USA

## Abstract

This report provides the whole-genome sequence of *Streptococcus agalactiae* isolate GB00037 isolated from a newborn in Calgary, Canada. This serotype V isolate is unique because it lacks pigment production previously shown to be critical for *S. agalactiae* virulence.

## GENOME ANNOUNCEMENT

*Streptococcus agalactiae*, also known as group B *Streptococcus* (GBS), is a leading cause of sepsis and meningitis in neonates worldwide. GBS typically has hemolytic activity and produces a yellow-orange pigment in culture, which are phenotypes encoded by genes within the *cyl* operon (1, 2). Pigment production is important for diagnostics and has been shown to be critical for virulence (3-6). However, not all invasive GBS strains are hemolytic and a small proportion lack pigment (7); hence, the mechanism of pathogenesis in these strains is likely due to other virulence factors. Strain GB00037 was recovered from the blood of a septic neonate with early onset disease in Calgary, Canada in 2000 (8). GB00037 is a serotype V, nonpigmented and nonhemolytic strain that was classified as multilocus sequence type (ST)-1. Genome analysis revealed an intact *cyl* operon, and therefore, additional phenotypic and sequencing analyses are warranted to identify the genes and pathways required for pathogenesis. Because GB00037 represents an atypical invasive strain, the genome is an important addition to GenBank.

For sequencing, genomic DNA was extracted and purified using the UltraClean microbial DNA isolation kit (MO BIO Laboratories, Inc., Carlsbad, CA), and sequencing was performed using an Illumina MiSeq (Illumina Inc., San Diego, CA) with a 500 cycle, paired-end 250 post library preparation using the Illumina Nextera XT kit. Post ambiguous sequences and adapters were trimmed with Trimmomatic (9) followed by quality checking using FastQC (http://www.bioinformatics.babraham.ac.uk/projects/fastqc/) and assembly with Velvet 1/2/07 (10) resulting in 34× coverage.

Annotation of the 2,045,700 bp draft genome was performed using the Prokaryotic Genomes Annotation Pipeline (http://www.ncbi.nlm.nih.gov/genome/annotation_prok/). Annotated features include 2,159 genes with 2,117 coding sequences (CDS), 3 rRNAs, 16 tRNAs, and 1 noncoding RNA (ncRNA). Functional annotation using with the Rapid Annotation using Subsystem Technology (RAST) Server (11) identified 1,988 coding sequences with 19 RNAs. Furthermore, 56% of the genes covered subsystem features and 67 of these genes were associated with virulence, while 16 genes were phage-associated. Many genes (*n* = 259) were linked to carbohydrates and carbohydrate metabolism, protein metabolism (*n* = 177), and cell wall and capsule (*n* = 141). The Resistance Gene Identifier (RGI) in the Comprehensive Antibiotic Resistance Database (12) identified 10 genes conferring resistance to fluoroquinolones (*n* = 2), β-lactams (*n* = 5), peptides (*n* = 1), a tetracycline derivative (*n* = 1), and multidrug resistance to macrolides and lincosamides (*n* = 1).

### Nucleotide sequence accession numbers.

This whole-genome shotgun project has been deposited at DDBJ/EMBL/GenBank under the accession no. LGAH00000000. The version described in this paper is version LGAH01000000.
